# Integrating single-nucleus RNA sequencing and gene perturbation reveals regulatory networks of intramuscular fat deposition in bovine skeletal muscle

**DOI:** 10.5713/ab.250897

**Published:** 2026-03-11

**Authors:** Fengying Ma, Mingjuan Gu, Lin Zhu, Risu Na, Caixia Shi, Guiting Han, Chencheng Chang, Le Zhou, Yanchun Bao, Shuai Li, Yaqiang Guo, Jiaxin Zhang, Wenguang Zhang

**Affiliations:** 1 College of Animal Science, Inner Mongolia Agricultural University, Hohhot, China; 2 College of Life Science, Inner Mongolia Agricultural University, Hohhot, China

**Keywords:** BMPR1A, Gene Perturbation, Intramuscular Fat, Single-nucleus RNA Sequencing, Vascular Endothelial Cells

## Abstract

**Objective:**

Intramuscular fat (IMF) is key for meat quality. Although fibro/adipogenic progenitor cells (FAPs) participate in adipogenesis, roles of other stromal cells remain unclear. This study characterized cellular heterogeneity in cattle IMF deposition and identified essential cell types and mechanisms regulating intramuscular adipogenesis.

**Methods:**

We performed single-nucleus RNA sequencing on longissimus dorsi muscle samples from three cattle breeds (Angus, Hereford, and Simmental) with divergent IMF content. Cell populations were annotated using multiple reference-based methods. We applied Scissor to identify phenotype-associated cells, CellChat to infer intercellular communication, high-dimensional weighted gene co-expression network analysis to detect co-expression modules, and single-cell flux estimation analysis to evaluate metabolic activity. Virtual gene perturbation was evaluated using scTenifoldKnk, and drug-responsive cell subpopulations were assessed with scRank. Additional *in vitro* validation experiments were conducted to confirm the regulatory effect of *BMPR1A*.

**Results:**

Ten cell types were identified. Angus (high marbling) showed elevated proportions of adipocytes, FAPs, and vascular endothelial cells (VEndoCs), while Hereford were enriched in type II fibers. VEndoCs were consistently linked to high IMF, acting as central signaling hubs that interact extensively with adipocytes and FAPs, and exhibited high glucose metabolic activity. Co-expression analysis within VEndoCs revealed 40 candidate regulators including nine BMP signaling pathway genes. Virtual knockdown identified *BMPR1A* as top regulator, altering expression of 13 lipid metabolism-related genes. *In vitro* validation confirmed that *BMPR1A* inhibition downregulated *PPARγ* mRNA, validating its positive regulatory role in intramuscular adipogenesis. Neurocytes showed highest sensitivity to resveratrol (via *ALDH2*), whereas FAPs were least responsive.

**Conclusion:**

Our findings indicate VEndoCs actively regulate intramuscular adipogenesis via a BMPR1A-mediated signaling pathway, supported by *in vitro* validation. These results provide insights into the cellular and molecular basis of marbling in cattle, highlighting *BMPR1A* as a potential target for genetic or nutritional strategies to improve meat quality.

## INTRODUCTION

Intramuscular fat (IMF) deposition is a critical determinant of meat quality in beef cattle, significantly influencing key sensory attributes such as tenderness, juiciness, and flavor [1]. Beyond its role in palatability, IMF—visually appreciated as marbling—also contributes to aroma development and texture during cooking, thereby enhancing overall eating quality and commercial value. IMF constitutes a specialized depot of adipose tissue embedded within skeletal muscle, and its progressive accumulation is governed by adipogenesis—the biological process by which intramuscular adipogenic precursors differentiate and mature into lipid-filled adipocytes. In many production systems, cull cows represent a substantial portion of the beef supply; however, advanced age, metabolic stress, and extended lactation often result in reduced IMF accumulation and consequently inferior meat quality [2]. Consequently, beef derived from cull cows often demonstrates reduced palatability and lower grading, limiting its use in high-value market segments. In global, where heifer culling rates exceed 10%, beef from culled cattle exhibits significantly lower IMF content compared to beef from feedlot-fattened cattle, resulting in reduced sensory quality and consumer acceptance [3]. Strategic nutritional interventions during the 90–120 days pre-slaughter have therefore gained attention as a viable approach to enhance marbling in cull cows [4]. These regimens often include high-energy diets, dietary lipid supplementation, and metabolic modifiers aimed at shifting nutrient partitioning toward intramuscular adipogenesis.

The deposition of IMF is governed by complex interactions between adipogenic progenitors, myofibers, and the vascular niche, which collectively influence meat quality [5]. Although genomic studies have identified candidate regions and genes associated with fat deposition traits, these findings largely reflect population-level associations rather than cell-type-specific mechanisms [6]. Transcriptomic and metabolomic studies have highlighted genes such as *PPARγ*, *FABP3*, and *LEPR* as regulators of lipid metabolism and adipogenesis in beef cattle, yet they fall short of elucidating the spatial orchestration of lipid storage within the muscle microenvironment [7]. Growing evidence suggests that adipogenic precursors engage in paracrine crosstalk with endothelial and satellite cells [8], but the hierarchy of these interactions remains unclear—especially in postmortem muscle, where cellular states influence lipid crystallization and meat quality [9]. Importantly, most mechanistic insights originate from murine models or human clinical studies [10], which may not fully capture species-specific adaptations in bovine muscle, a tissue characterized by distinct metabolic and developmental trajectories.

To address this knowledge gap, we applied single-nucleus RNA sequencing (snRNA-seq) to characterize cellular heterogeneity and identify molecular drivers of IMF deposition in three cattle breeds with divergent fattening potentials: Angus (ANG), Hereford (HFD), and Simmental (SIM). ANG cattle are recognized for high marbling ability, SIM for lean growth efficiency, and HFD represent an intermediate phenotype [11,12]. These breeds serve as ideal models for dissecting cellular mechanisms underlying IMF variation.

Recent advances in snRNA-seq have revolutionized the study of complex tissues by enabling the identification of rare cell types, delineation of differentiation trajectories, and inference of cell-cell communication networks [13]. In skeletal muscle, this technology has revealed the functional diversity of fibro/adipogenic progenitor cells (FAPs), endothelial subsets, and immune cells, providing new insights into the regulation of adipogenesis and muscle homeostasis [14].

In this study, we hypothesize that vascular endothelial cells (VEndoCs) serve as active regulators of IMF deposition through BMP-signaling-mediated crosstalk with adipogenic precursors. By integrating snRNA-seq data with *in silico* perturbation and pharmacological validation, we aimed to uncover breed-specific regulatory programs and identify key candidate genes controlling intramuscular adipogenesis. Our findings not only provide novel insights into the cellular basis of marbling but also offer potential targets for genetic and nutritional strategies aimed at improving meat quality in beef cattle.

## MATERIALS AND METHODS

### Animals and tissue processing

*Longissimus dorsi* (LD) muscle tissues were collected from 4-year-old female ANG (n = 2), HFD (n = 2), and SIM (n = 2) cattle raised under standardized conditions at Xuyi Animal Husbandry. All cattle were individually penned and fed a standardized high-energy finishing diet for 180 days prior to sampling to minimize environmental and nutritional variation. The total mixed ration (TMR) consisted of (on a dry matter basis): 60% whole-plant corn silage as the primary roughage source, 35% concentrated supplement (including steam-flaked corn, soybean meal, wheat bran, and a mineral-vitamin premix), and 5% supplemental hay. The diet was formulated to meet or exceed nutrient requirements for beef cattle finishing. Animals had ad libitum access to feed and fresh water. All animals were fed identical diets to minimize nutrition-related confounding factors. For RNA integrity preservation, samples were flash-frozen in liquid nitrogen within 5 min of collection and stored at −80°C until further processing.

### Single nucleus suspension preparation and sequencing

For the preparation of single-nucleus suspensions, thawed tissue samples were minced and homogenized in a Dounce homogenizer (TIANDZ) with 1 mL of ice-cold homogenization buffer (250 mM sucrose, 10 mg/mL BSA, 5 mM MgCl_2_, 0.12 U/μL RNasin Plus [Promega, N2115], and 1× complete Protease Inhibitor Cocktail [Roche, 11697498001]) using 25–50 gentle strokes with a loose pestle. The homogenate was filtered through a 40 μm strainer and centrifuged at 500×g for 5 min at 4°C. The pellet was resuspended in nuclear suspension buffer (320 mM sucrose, 10 mg/mL BSA, 3 mM CaCl_2_, 2 mM magnesium acetate, 0.1 mM EDTA, 10 mM Tris-HCl, 1 mM DTT, 1× protease inhibitor, and 0.12 U/μL RNasin Plus) and adjusted to a final concentration of 1,000 nuclei/μL.

Single-nucleus libraries were constructed using the DNBelab C Series High-throughput Single-cell RNA Library Preparation Set V2.0. Briefly, suspensions were encapsulated into droplets, followed by bead-based mRNA capture, reverse transcription, cDNA amplification, and purification. The resulting barcoded cDNA and oligonucleotide libraries were pooled and sequenced on the DNBSEQ-T7 platform with a PE100 strategy. A total of six libraries (two per breed) were sequenced. To ensure the reliability of downstream analysis, we calculated key sequencing metrics: the average sequencing depth was approximately 64k reads per nucleus, and the median number of genes detected per high-quality nucleus was 1,153 (range: 1,051–1,404). High-quality nuclei were defined as those with detected genes≥200, UMI counts≥500, and mitochondrial gene fraction<5% (consistent with the QC criteria described in the Sample Preparation section). These metrics confirm that the sequencing depth and gene detection efficiency are sufficient for accurate cell type annotation and transcriptional profile analysis. Post-sequencing quality assessment led to the exclusion of one SIM library due to inadequate quality, resulting in five high-quality libraries being retained for all subsequent analyses.

### Quality control and preprocessing of data with single-nucleus RNA sequencing

To preprocess and perform quality control on the snRNA-seq data, the Bos taurus reference genome (Bos_taurus.ARS-UCD1.2.dna.toplevel.fa) and corresponding annotation file (Bos_taurus.ARS-UCD1.2.110.gtf) were retrieved from the ENSEMBL database. A genome index was constructed using the “DNBC4tools mkref” command. Prior to cell-level preprocessing, stringent library-level quality control was performed to exclude low-quality libraries using DNBC4tools (v2.1.0) and Seurat (v4.4.1). Ambient RNA contamination was eliminated with SoupX (v1.6.2) under default settings [15]. Potential doublets were identified and removed using DoubletFinder (v2.0.3) [16], also using default parameters. Additional filtering steps included the removal of cells with fewer than 200 detected genes or mitochondrial gene content exceeding 15%, as well as genes detected in fewer than three cells. The gene expression data were normalized using the “NormalizeData” function in Seurat. Highly variable genes were identified with “FindVariableFeatures” (selection.method = “vst”, nfeatures = 2,000) for downstream dimensional reduction. To alleviate the issue of batch effects, we evaluated four batch correction methods: ComBat, scVI, Harmony, and principal component analysis (PCA). We use the following metrics to evaluate their performance: isolated label F1 score (evaluating batch mixing), KMeans NMI and ARI (evaluating inter batch biological protection), and average contour width (evaluating cluster compactness). Supplement 1 summarizes the performance of each method on these indicators. ScVI achieved the best overall performance and was therefore used to integrate datasets of all three varieties before downstream analysis. The integrated data were then scaled, and PCA was performed. Cell clustering was conducted using the “FindClusters” function with resolution parameters of 0.4, which was selected based on an evaluation of clustering robustness and biological interpretability. Finally, nonlinear dimensional reduction was carried out using RunUMAP for visualization.

### Cell type annotation

A three-tiered cell type annotation strategy was employed to ensure accurate and robust classification: i) Preliminary annotation was performed using scMayoMap, an automated single-cell annotation tool [17]; ii) The identity of each cluster was determined by combining differentially expressed gene (DEG) sets identified through cell cluster differential analysis with typical cell type markers; and iii) Probabilistic cell typing was conducted by integrating a priori knowledge of marker genes through a statistical model to assign identities to both predefined and de novo cell populations [18].

### Phenotypic correlation analysis

Public bulk RNA-seq datasets (GSE186730 [19],GSE171876 [20]) were normalized (DESeq2 v1.38.0) and integrated with snRNA-seq data using Harmony (v0.1.1) to correct batch effects. Scissor (v1.0.4) [21] identified phenotype-associated cells (parameters: α = 0.05, family = “gaussian”) by correlating bulk-sample similarity matrices with meat quality traits. Cells with Scissor FDR<0.01 were classified as “Scissor+”.

### Analysis of cellular communication networks

CellChat [22], an open-source R package ( https://github.com/sqjin/CellChat , accessed on 1 July 2022), inferred intercellular signaling networks using ligand-receptor pairs from HPRD, DIP, and MSigDB databases. Signaling probabilities were computed via permutation tests (n = 1,000), prioritizing pathways with differential activity across breeds (|log2FC|>1, p<0.01).

### High-dimensional weighted correlation network analysis

We performed high-dimensional weighted gene co-expression network analysis (hdWGCNA) [23] to establish co-expression networks from single-cell resolution data using established computational methods. Our analytical pipeline involved: (1) filtering genes detected in ≥5% of cells as input features; (2) generating metacell expression matrices via MetacellsByGroups; (3) optimizing soft-thresholding parameters using TestSoftPowers; (4) constructing final networks with ConstructNetwork. Hub genes within each module were identified based on eigengene-based connectivity (kME), which quantifies the correlation between individual gene expression and the module eigengene. Genes with higher kME values represent more central components of the co-expression module. All procedures strictly followed published protocols.

### Gene virtual knockout

ScTenifoldKnk (v1.0.1) [24] is a computational framework designed to perform *in silico* gene knockout (KO) experiments using snRNA-seq data the workflow begins by constructing a single-cell gene regulatory network (scGRN) from wild-type (WT) expression matrices through tensor decomposition and manifold alignment, capturing gene-gene interactions at cellular resolution. To simulate KO effects, the target gene is virtually removed by setting its outgoing edges to zero in the scGRN, generating a pseudo-KO network. Differential regulatory impacts are then quantified by comparing the perturbed network against the original WT network using quasi-manifold alignment algorithms, which identify globally and locally disrupted interactions. This approach enables systematic assessment of gene function without requiring experimental KO models, leveraging snRNAseq-seq data to infer cell-type-specific regulatory dynamics.

### Cell culture

The bovine skeletal muscle microvascular endothelial cells were purchased from Cellverse. The culture medium consisted of 93% bovine skeletal muscle microvascular endothelial cell specific medium, 5% fetal bovine serum, 1% bispecific antibody, and 1% primary endothelial cell culture additive. When the cells reached 70%–80% fusion, they were divided into three groups: control group (untreated), 0.1% DMSO group, and Dorsomorphin treatment group (treated with 2.5 μM Dorsomorphin, a specific *BMPR1A* inhibitor purchased from MCE company). Each group is assigned three biological replicates and three technical replicates. After 24 hours of processing, collect cells for subsequent experiments.

### Western blot

Total protein was extracted from cells using RIPA lysis buffer (containing protease inhibitor, Beyotime). Measure protein concentration using BCA protein detection kit (Beyotime). Separate equal amounts of protein (30 μg per lane) using 10% SDS-PAGE and transfer them onto a PVDF membrane. After blocking with 5% skim milk at room temperature for 1 hour, the membrane was incubated overnight at 4°C with primary antibodies against *BMPR1A* (1:1,000), *PPARγ* (1:1,000), and *GAPDH* (internal reference) (1:5,000, Beyotime). Then, the membrane was incubated with a horseradish peroxidase (HRP) conjugated secondary antibody (1:5,000, Beyotime) at room temperature for 1 hour. Visualize protein bands using ECL chemiluminescence assay kit (Beyotime) and quantify band grayscale values using ImageJ software.

### Quantitative real-time polymerase chain reaction

Total RNA was extracted from cultured cells using Vazyme Zol Reagent (Vazyme RC202). The purity and concentration of the extracted RNA were determined using a NanoDrop 2000 spectrophotometer (Thermo Fisher Scientific), and only RNA samples with an A260/A280 ratio of 1.8–2.1 were used for subsequent experiments. First-strand cDNA was synthesized from 1 μg of total RNA using the HiScript IV 1st Strand cDNA Synthesis Kit (Vazyme R412) following the manufacturer’s instructions. Quantitative real-time polymerase chain reaction (qRT-PCR) was performed using the ChamQ Universal SYBR qPCR Master Mix (Vazyme Q711) on a CFX96 Real-Time PCR System (Bio-Rad) to detect the relative expression levels of *BMPR1A* and *PPARγ*. *GAPDH* was used as the internal reference gene, and the 2^^−ΔΔCt^ method was applied to calculate the relative mRNA expression levels between the control and treatment groups. All reactions were performed in three biological replicates and three technical replicates. The specific primer sequences for target and reference genes are listed in Table 1, and all primers were synthesized by Sangon Biotech.

### Network pharmacology

ScRank (v1.0.0) [25] is a computational tool based on the target perturbation gene regulatory network (tpGRN), aimed at inferring drug responsive cell types from snRNA-seq seq data that has not been treated with drugs. The core idea is to simulate drug inhibition effects by removing nodes and associated edges of drug target genes in the gene regulatory network (GRN), and then use manifold alignment algorithm and network diffusion model to quantify the differences between the original GRN and the perturbed tpGRN. The larger the difference, the more sensitive the cell type is to drugs.

### Metabolic fluxomes and abundances analysis

Single-cell flux estimation analysis (scFEA) (v1.1.2) [26] is a computational method for inferring cellular metabolic fluxomes and metabolite abundances from snRNA-seq data using flux balance constraints based on novel probabilistic models. All cell types were calcuated the metabolic Fluxomes and Abundances by scFEA in python. After calculating metabolic flux and metabolite abundances, the 70 metabolic modules and all metabolites were selected for visualization by heatmap.

### Functional enrichment analysis

Key modules were analyzed via clusterProfiler (v4.8.1) against Kyoto Encyclopedia of Genes and Genomes (KEGG) and Gene Ontology (GO) databases. Terms with q-value<0.05 (Benjamini-Hochberg correction) were considered significant. Protein-protein interaction (PPI) networks were visualized using STRING (v12.0) and Cytoscape (v3.9.1).

## RESULTS

### Single-nucleus RNA sequencing reveals breed-specific cellular heterogeneity in longissimus dorsi

**Figure 1:**
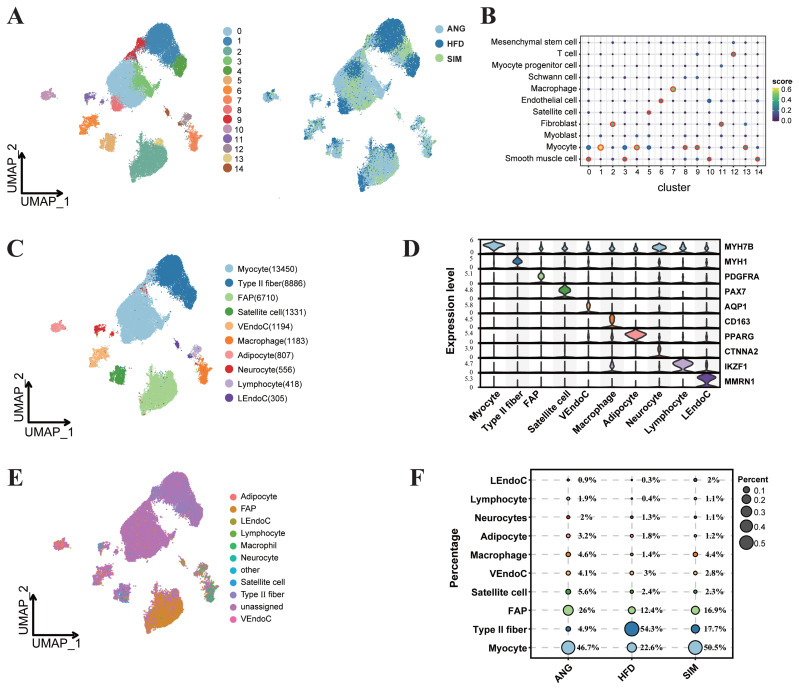
Single-nuclei RNA sequencing reveals cellular heterogeneity in skeletal muscles of different bovine breeds. (A) UMAP graphs show unsupervised clustering of cells from all breeds identified by snRNA-seq. (B) A broad preliminary automatic cell type annotation using scMayoMap. (C) The final refined annotation based on canonical marker genes and published references. (D) Violin plots of relative expression of marker genes in 10 cell types. (E) UMAP projection showing results of cellassign annotations. (F) Dot plot showing the proportions of different species cell types. ANG, Angus; HFD, Hereford; SIM, Simmental; snRNA-seq, single-nucleus RNA sequencing.

To characterize the cellular composition of bovine skeletal muscle across breeds with divergent marbling potential, we collected LD muscle from ANG, HFD, and SIM cattle. Using droplet-based snRNA-seq, we captured a total of 43,398 high-quality nuclei, with breed-specific distributions of 16,582 (ANG), 15,736 (HFD), and 7,177 (SIM). Following data integration and stringent quality filtering, 15,315 (ANG), 12,777 (HFD), and 6,748 (SIM) cells were retained for subsequent analysis. Unsupervised clustering using UMAP revealed 15 distinct cell clusters common to all breeds (Figure 1A).

We employed a sequential strategy for cell type annotation. An initial broad classification using scMayoMap (FDR< 0.05) assigned cells into seven major categories, including myoblasts and satellite cells (Figure 1B). Subsequent refinement using well-established lineage-specific markers enabled the identification of 10 finely resolved cell types: Myocytes (*CKM*), Type II fibers (*MYH1*), FAPs (*PDGFRA*), Satellite cells (*PAX7*), VEndoCs (*AQP1*), Macrophages (*CD163*), Adipocytes (*PPARΓ*), Neurocytes (*CTNNA2*), Lymphocytes (*IKZF1*), and Lymphatic Endothelial Cells (LEndoCs, *MMRN1*) (Figure 1C), consistent with previous studies [14,27–30]. Violin plots confirmed the cluster-specific expression of these marker genes (Figure 1D). To further support annotation robustness, we performed probabilistic cell typing using Cellassign (FDR<0.1). This conservative reference-based method validated the annotation of nine cell types with high confidence (Figure 1E). Notably, Cellassign did not fully resolve myocyte subpopulations, likely due to their highly similar transcriptional profiles and overlapping marker expression rather than distinct canonical cell identities. Importantly, our final annotation of 10 cell types was based on consensus evidence from multiple complementary approaches, including marker gene expression, published references, functional enrichment, and multiple annotation tools, rather than relying solely on Cellassign.

An analysis of cellular composition across breeds revealed notable differences (Figure 1F). The ANG samples contained the highest proportions of myocytes (46.7%) and FAPs (26.0%), which together constituted 72.7% of all nuclei. In contrast, the HFD samples were characterized by a predominance of Type II fibers, which comprised for 54.3% of cells, a proportion 11.1-fold and 3.1-fold higher than that observed in ANG (4.9%) and SIM (17.7%), respectively. SIM samples exhibited elevated macrophages (4.4%) and LEndoCs (2.0%) populations, alongside a lower percentage of FAPs (16.9%) compared to ANG. The detailed individual consistency analysis is presented in Supplement 2.

To explore the potential contribution of myoblast transdifferentiation to IMF deposition, we identified *PPARΓ^+^/MYOD1^+^* double-positive cells (transdifferentiation intermediates) from the snRNA-seq dataset. A total of 20 such cells were detected (0.052% of total nuclei) with 4 in ANG, 4 in HFD and 12 in SIM, and chi-square test confirmed no significant interbreed distribution difference (p>0.05). All these cells showed 100% co-expression of *PPARγ* and *MYOD1*, with gradient expression of downstream myogenic (*CKM*, *MYH1*) and adipogenic (*FABP4, ACACB*) markers (Supplements 3, 4), confirming their identity as genuine myogenic-adipogenic transition-state cells.

### Integration of single-nucleus RNA sequencing and Bulk RNA-seq to identify candidate cell types

**Figure 2:**
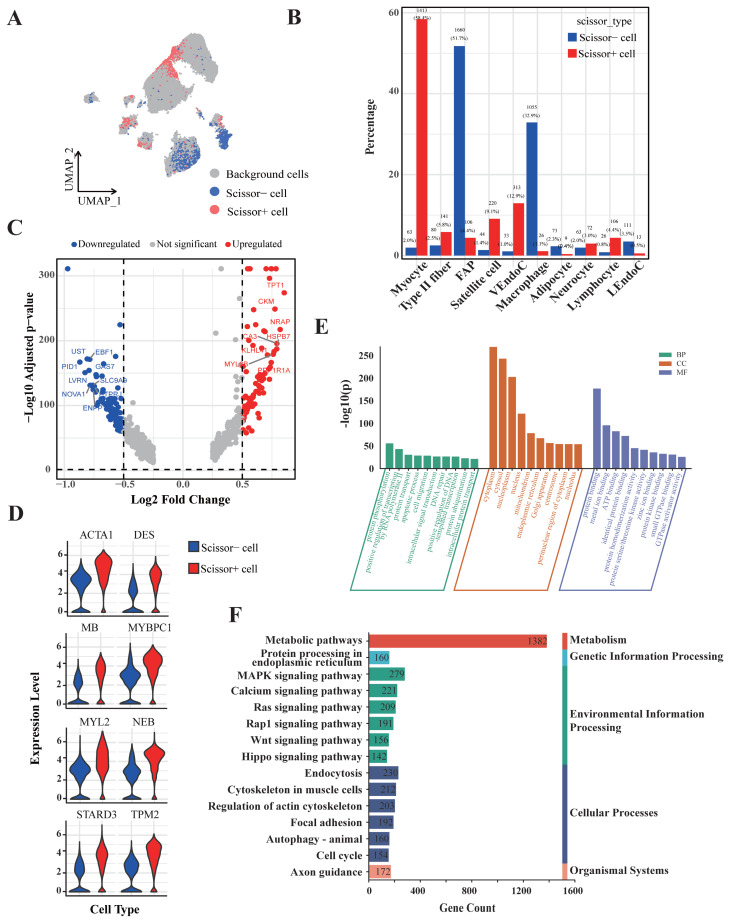
Scissor joint analysis to identify candidate cell types. (A) UMAP visualization of Scissor-selected cells. Red and blue dots are Scissor^+^ (Scissor_H, high IMF associated cells) and Scissor- cells (Scissor_L, low IMF associated cells). (B) Distribution and proportion of Scissor^+/−^ cells within each identified cell cluster. (C) Volcano plot of differential gene expression in Scissor^+^ cells vs. Scissor^−^ cells. Red and blue dots have genes with significantly increased or decreased expression in Scissor^+^ cells (–Log10 Adjusted p-value>0, absolute Log2 fold change>0.5). (D) Violin plot of expression levels of upregulated genes by top8. (E) Gene Ontology (GO) enrichment analysis of genes upregulated in IMF-associated Scissor^+^ cells. (F) Kyoto Encyclopedia of Genes and Genomes (KEGG) pathway analysis highlighting functional pathways enriched in Scissor^+^ cells. IMF, intramuscular fat.

To identify cell types associated with IMF deposition, we analyzed bulk RNA-seq data from LD with high IMF (ANG, H-group) and low IMF (SIM, L-group). To integrate these bulk transcriptomic profiles with our snRNA-seq dataset, we employed the Scissor algorithm. This computational analysis identified 5,124 cells whose transcriptional profiles were significantly associated with the marbling phenotype, among which 1,859 showed a correlation with high IMF (designated Scissor_H cells) and 3,265 with low IMF (Scissor_L cells) (Figure 2A). According to this analysis, Scissor_H cells were primarily classified as myocytes, VEndoCs, and satellite cells (Figure 2B). To explore the transcriptional underpinnings associated with the high-IMF phenotype, we compared gene expression profiles between Scissor_H and Scissor_L cells and identified 86 up-regulated and 92 down-regulated DEGs (Figure 2C). Expression patterns of eight representative up-regulated genes are illustrated in Figure 2D. GO enrichment analysis indicated that DEGs up-regulated in Scissor_H cells were enriched for biological processes such as protein phosphorylation, positive regulation of transcription by RNA polymerase II, and protein transport (Figure 2E). KEGG pathway enrichment analysis further revealed significant enrichment of DEGs in metabolic pathways, endoplasmic reticulum protein processing, MAPK signaling, calcium signaling, Wnt signaling, and Hippo signaling pathways (Figure 2F), suggesting a potential role for these processes in the high-IMF phenotype.

**Figure 3:**
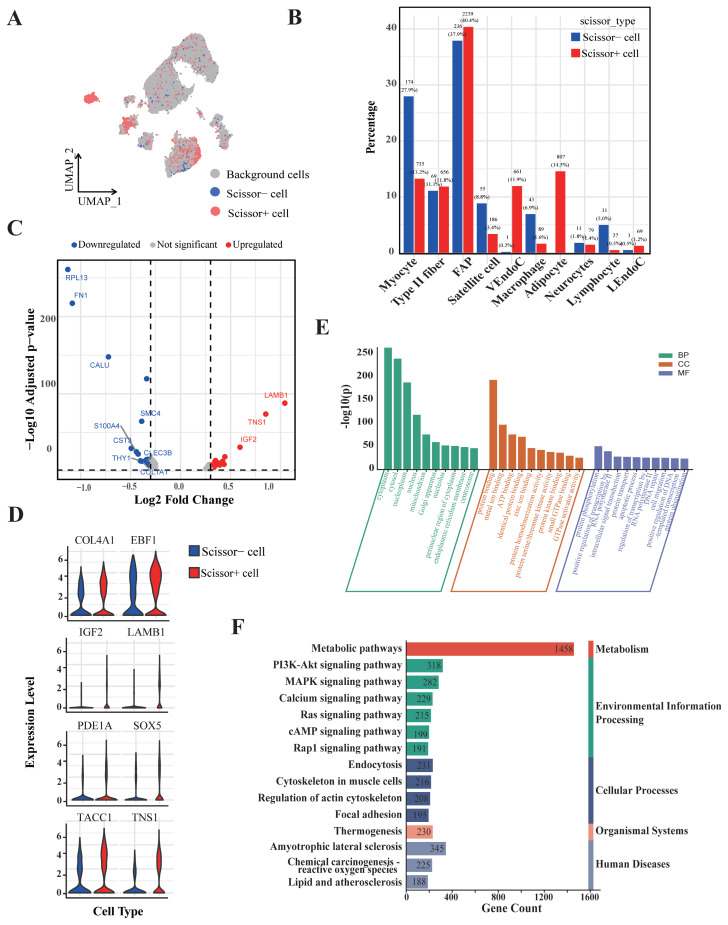
Scissor co-analysis to identify candidate cell types. (A) UMAP visualization of Scissor-selected cells. Red and blue dots are Scissor^+^ (Scissor_P, resveratrol-treated group) and Scissor^−^ (Scissor_C, control group) cells, respectively. (B) Cluster composition of Scissor^+/−^ cells showing the proportion contributed by each cell type. (C) Comparative volcano plot of differentially expressed genes between Scissor^+^ and Scissor^−^ populations. Genes significantly upregulated or downregulated in Scissor^+^ cells are highlighted in red and blue. (D) Violin plot of expression levels of upregulated genes by top8. (E) GO functional enrichment analysis of genes induced in resveratrol-associated Scissor^+^ cells. (F) KEGG pathway analysis of upregulated genes to reveal signaling programs activated in Scissor^+^ cells. GO, Gene Ontology; KEGG, Kyoto Encyclopedia of Genes and Genomes.

To further investigate candidate cell identified as associated with superior marbling traits and to explore their potential mechanistic responses using an independent perturbation model, we turned to resveratrol, a natural polyphenol previously demonstrated to enhance IMF deposition through dietary supplementation. We obtained RNA-seq data from resveratrol-treated and untreated control bovine primary myocytes and integrated these with our snRNA-seq dataset using the Scissor framework. This approach enabled the identification of muscle cell subpopulations exhibiting transcriptional responses consistent with resveratrol-induced adipogenic regulation. Among the 6,171 Scissor-selected cells, 5,548 Scissor+ cells were associated with the resveratrol-treated group (designated Scissor_P cells), while 623 Scissor- cells corresponded to the control group (Scissor_C cells). Adipocytes and VEndoCs were identified as the primary sources of Scissor_P cells (Figures 3A, 3B). To delineate the transcriptional profiles underlying the adipogenic response, we compared gene expression between Scissor_P and Scissor_C cells, identifying 26 up-regulated and 20 down-regulated DEGs (Figure 3C). The expression patterns of eight representative up-regulated genes are displayed in Figure 3D. Functional enrichment analysis indicated activation of processes associated with cytoplasmic, cytosolic, and nucleoplasmic compartments in Scissor_P cells (Figure 3E). KEGG pathway analysis further revealed that the up-regulated DEGs were significantly enriched in metabolic pathways, PI3K-Akt signaling, MAPK signaling, calcium signaling, cAMP signaling, and Rap1 signaling pathways (Figure 3F).

### Cellular communication analyzes interactions between key cell types

**Figure 4:**
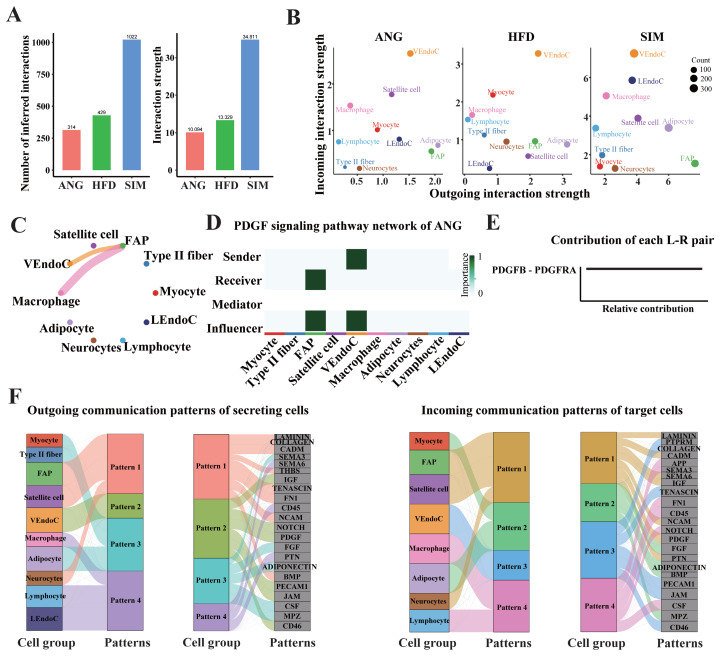
Comparison of cell communication and signaling pathways in three species. (A) Bar graph of the number of cellular interactions for the three species. The left graph shows the number of interactions and the right graph shows the strength of interactions. (B) Dot plots showing the interaction strength of incoming (y-axis) and outgoing (x-axis) signaling in each cell types. The circle size indicates the interaction number in the specified cell type with other cell types. (C) Chord diagram of the PDGF pathway of ANG cattle. Lines represent intercellular interactions, and their thickness is proportional to the communication probability of the cell interaction. Line colors are consistent with the signaling source. (D) Heatmap shows the role (sender, receiver, mediator or influencer) of each cell group in PDGF signaling of ANG cattle. Color saturation indicates the importance of signaling role in the pathway network. (E) Relative contribution of ligand-receptors of the PDGF pathway to the overall signaling network. (F) The outgoing communication patterns and incoming communication patterns of secreting cells of ANG cattle. The flow thickness represents the contribution of a signaling pathway to each mode of communication. ANG, Angus; HFD, Hereford; SIM, Simmental.

Scissor analysis based on two independent phenotype comparisons consistently identified VEndoCs as a dominant positively correlated cell type. This convergence result provides a strong reason for using VEndoCs as the core regulatory cell type. To further describe their functional roles, we performed a comprehensive CellChat analysis on snRNA-seq datasets derived from ANG, HFD, and SIM cattle. With ligand-receptor interactions predicted based on transcriptomic abundance of ligands and their cognate receptors, it should be noted that these interactions represent putative signaling events and do not confirm direct functional protein-level signaling or downstream biological effects. Our analysis identified extensive and breed-specific interaction networks, comprising 314, 420, and 1,022 significant ligand–receptor-mediated interactions (p≤0.05), respectively (Figure 4A). Several key signaling pathways, including PTPRM, BMP, IGF, NOTCH, PDGF, TGFβ, VEGF, and FGF were found to be active across the ten annotated cell clusters. Based on network centrality measures, FAPs, adipocytes, and VEndoCs emerged as dominant signaling hubs, serving as major senders and receivers within these communication networks (Figure 4B). Notably, no significant adipogenesis-related ligand-receptor interactions (e.g., BMP, IGF, PDGF signaling) were detected between myoblasts and FAPs across all three breeds, confirming that FAP adipogenic differentiation is not dependent on myoblast-derived paracrine signals.

Given the established importance of PDGF signaling in muscle development, we further dissected its inferred role across the three breeds. In the ANG network, FAPs were predicted to function as the primary targets of PDGF signaling, with communication being exclusively mediated by the PDGFB–PDGFRA ligand–receptor pair (Figures 4C–4E). In the HFD network, FAPs not only maintained PDGF responsiveness but also were inferred to acquire the ability to secrete PDGF ligands. Multiple cell types including VEndoCs, macrophages, and FAPs themselves, were identified as potential PDGF sources, with signaling specifically facilitated through the PDGFC–PDGFRA pair (Supplements 5A–5C). In the SIM network, FAPs exhibited predicted PDGF signaling, satellite cells additionally appeared as signal senders, and two distinct ligand–receptor pairs were implicated (Supplements 5D–5F). Across all breeds, FAPs consistently expressed PDGF receptors. Notably, in HFD cattle, FAPs were implicated in an autocrine signaling loop, serving concurrently as both source and target of PDGF signals.

To explore the coordinated functions of diverse cell populations and signaling pathways during muscle development, we applied the pattern recognition module of CellChat. This computational analysis identified distinct breed-specific patterns in both outgoing and incoming signaling: four patterns were identified in ANG, two in HFD, and four in SIM cattle (Figure 4F, Supplement 6). These patterns reflect groups of signaling pathways that demonstrate coordinated activity shifts across breeds. Notably, in ANG cattle, outgoing signaling from VEndoCs was predominantly assigned to pattern 2, while incoming signaling to VEndoCs was predominantly assigned to pattern 3. Further inference delineated IGF, NOTCH, and PDGF as major autocrine pathways within VEndoCs, while PTPRM, COLLAGEN, and TENASCIN emerged as principal paracrine signaling routes.

### Single-cell flux estimation analysis

**Figure 5:**
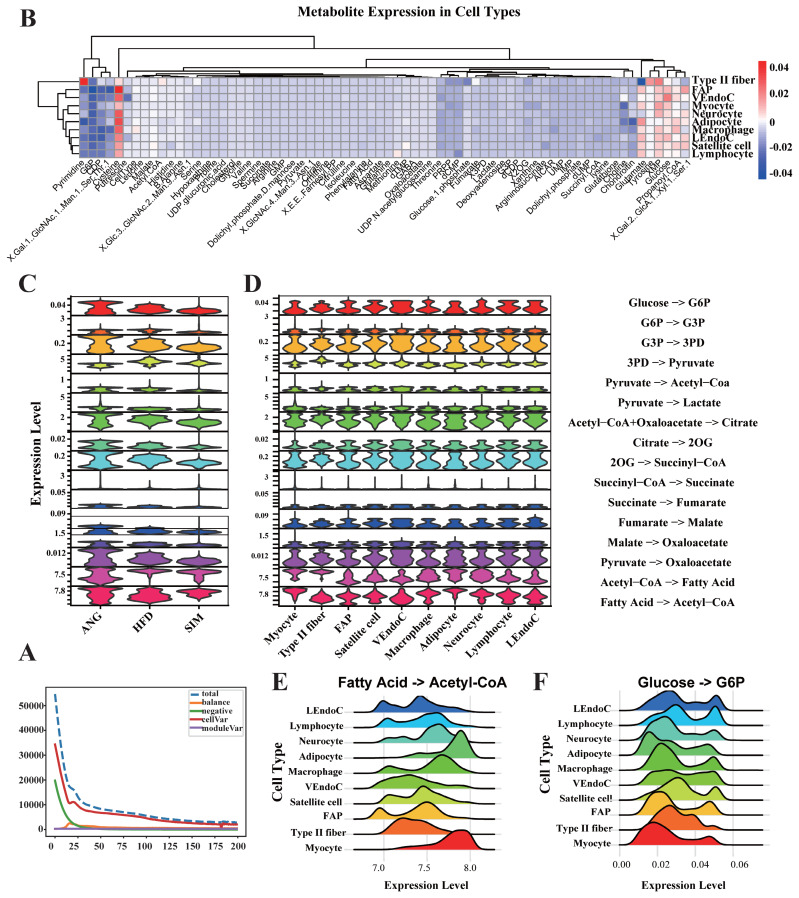
Comparison of metabolism landscape. (A) Convergence of the loss function during scFEA analysis. (B) Metabolite abundance of 10 cell types. (C) Violin plots of 16 modules enriched in 3 breeds. (D) Violin plots of 16 modules enriched in 10 cell types. (E) Ridge diagram of fatty acid to acetyl-CoA pathway expression in different cell types. (F) Ridge diagram of glucose to G6P pathway expression in different cell types. scFEA, single-cell flux estimation analysis; G6P, glucose-6-phosphate.

To assess metabolic heterogeneity among cell types in skeletal muscle, we applied scFEA on all cell subpopulations from the three cattle breeds. The convergence of the computational model was assessed via the loss function (Figure 5A). Analysis of metabolic aggregation based on UMAP embeddings revealed distinct patterns of enrichment: type II fibers displayed relatively strong activity in pyrimidine metabolism, FAPs and adipocytes were enriched in cysteine metabolism, and VEndoCs showed elevated glucose metabolism (Figure 5B).

We further evaluated the inferred activity of 16 metabolic modules associated with glycolysis, the TCA cycle, and fatty acid metabolism/synthesis across breeds and cell types. According to the scFEA model, ANG cattle exhibited higher overall enrichment of these pathways compared to HFD and SIM cattle (Figure 5C). At the cellular level, the model predicted that myocytes had the highest enrichment scores for conversions from succinyl-CoA to succinate and from pyruvate to lactate, whereas type II fibers showed the highest inferred activity for the conversion of 3-phosphoglycerate (3PG) to pyruvate and glucose-6-phosphate (G6P) to glyceraldehyde-3-phosphate (G3P) (Figure 5D). The total inferred activity across the 16 metabolic modules was computationally estimated to be significantly higher in satellite cells, VEndoCs, lymphocytes, and LEndoCs compared to other cell types. Consistent with this, the relative expression level associated with the conversion of fatty acids to acetyl-CoA was highest in adipocytes (Figure 5E), while the level associated with the conversion of glucose to G6P was highest in LEndoCs (Figure 5F).

### Identification of key gene modules and functional pathways associated with vascular endothelial cells using high-dimensional weighted gene co-expression network analysis

**Figure 6:**
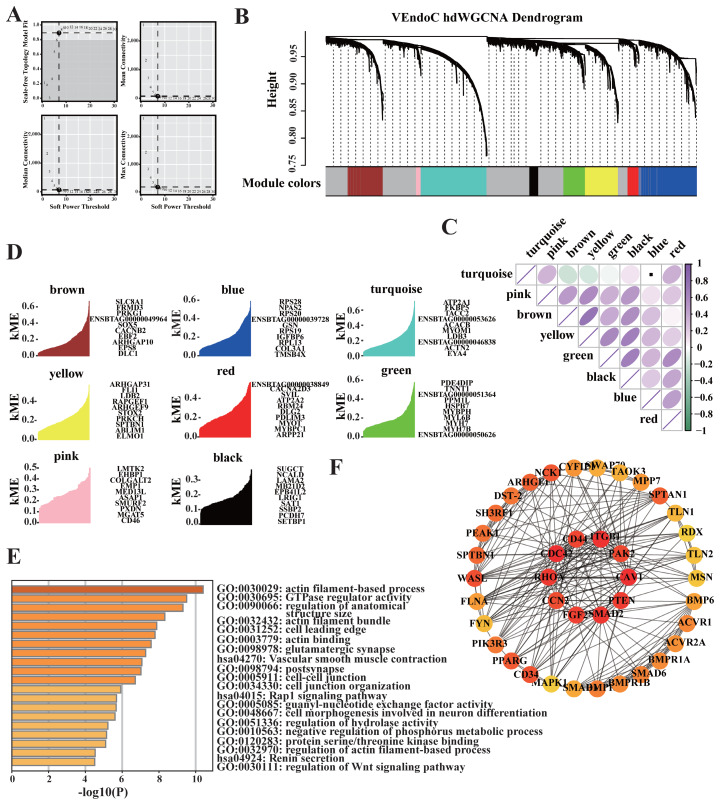
Identification of the crucial modules related to VEndoCs by hdWGCNA. (A) Select a soft power supply suitable for running the hdWGCNA, the mean value, median value and maximum connectivity of the topology network are shown respectively when different minimum soft thresholds are selected. (B) Construction of co-expression network using the optimal soft threshold of 7, with genes divided into 8 modules and resulting in a dendrogram. (C) Correlation analysis between the 8 modules. (D) KMEs for each module characterisation gene. (E) Barplot of gene enrichment items for key modules, colored by p-value. (F) Top40 gene interaction networks. VEndoCs, vascular endothelial cells; hdWGCNA, high-dimensional weighted gene co-expression network analysis.

To identify co-expression network within VEndoCs, we applied hdWGCNA. Using this method, a co-expression network was constructed with optimal connectivity, achieving a scale-free topological fitness index of 0.90 and a soft threshold power (β) of 7 (Figure 6A). This analysis identified eight distinct gene modules (Figure 6B), and their pairwise correlations were evaluated (Figure 6C). Based on metrics of module connectivity and correlation with cellular traits, the yellow and brown modules showed the strongest associations with VEndoCs clusters. Hub genes were defined as those with the highest eigengene-based connectivity (kME), a measure of correlation between gene expression and the module eigengene. The top 10 hub genes for each module are presented in Figure 6D. To functionally annotate the 926 genes comprising the yellow and brown modules, we performed PPI analysis using the STRING database. Subsequent enrichment analysis via Metascape suggested that these genes are computationally predicted to be involved in biological processes such as actin filament-based movement, GTPase regulator activity, regulation of anatomical structure size, actin filament bundle organization, cell leading edge, actin binding, glutamatergic synapse, and vascular smooth muscle contraction (Figure 6E). Furthermore, using the CytoHubba plugin in Cytoscape, we identified the top 40 hub genes within the PPI network (Figure 6F).

### Virtual perturbation validation of key genes and vitro validation

**Figure 7:**
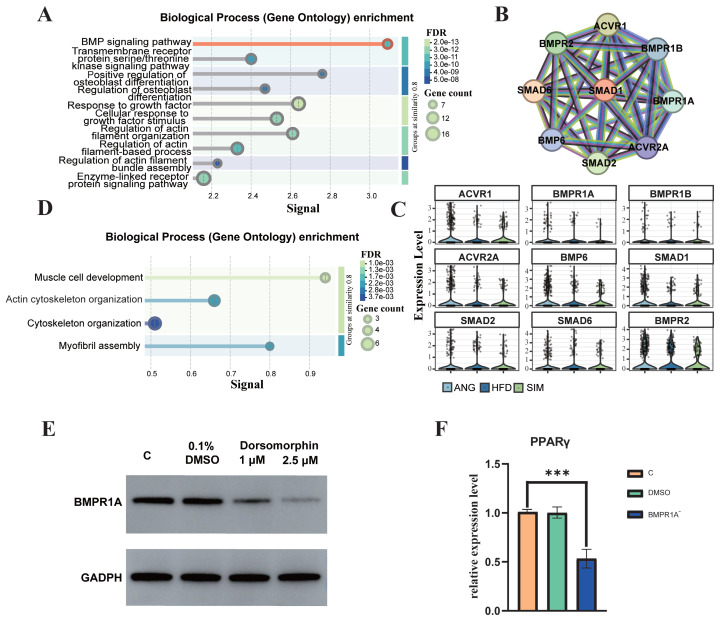
Validation of key genes. (A) The enrichment results of the top 40 genes identified by hdWGCNA. (B) The interaction of 9 genes involved in the BMP signaling pathway. (C) The expression of 9 genes involved in the BMP signaling pathway in 3 breeds. (D) Enrichment results of 13 genes affected by BMPR1A virtual knockout. (E) Western blot showed that Dorsomorphin inhibits BMPR1A protein expression. (F) qRT-PCR verification showed that the expression of PPARγ decreased after BMPR1A inhibition. hdWGCNA, high-dimensional weighted gene co-expression network analysis; qRT-PCR, quantitative real-time polymerase chain reaction.

From the 40 key genes identified through hdWGCNA, nine genes—*SMAD1*, *SMAD2*, *SMAD6*, *ACVR1*, *ACVR2A*, *BMP6*, *BMPR1A*, *BMPR1B*, and *BMPR2*—were found to be enriched in the BMP signaling pathway. We performed virtual knockdowns of these nine genes across VEndoCs from all three breeds (Figures 7A, 7B). Expression levels of these BMP pathway genes were highest in ANG cattle, as illustrated in Figure 7C. The computational perturbation analysis predicted that knockdown of *BMPR1A* would significantly alter the expression of 13 downstream genes. The model further suggested that silencing *ACVR1*, *ACVR2A*, *BMP6*, *BMPR2*, *SMAD1*, *SMAD2*, and *SMAD6* affected the expression of *MYH1* and *TNNC2* (adj. p-value<0.05). In contrast, the analysis predicted no significant gene expression changes following *BMPR1B* knockdown (Supplement 7). Subsequent pathway enrichment analysis of the genes predicted to be dysregulated after *BMPR1A* knockdown indicated a potential influence on biological processes related to muscle cell development and cytoskeletal organization, including actin cytoskeleton reorganization (Figure 7D).

To validate the virtual KO prediction results and bridge the logical gap between the key regulatory factor *BMPR1A* and the core adipogenic regulator *PPARγ*, we performed *in vitro* validation using primary bovine skeletal muscle endothelial cells. Cells were divided into three groups: blank control, 1% DMSO solvent control, and Dorsomorphin (2.5 μM) treatment group, with three biological replicates and three technical replicates per group. We first verified the inhibitory effect of Dorsomorphin on *BMPR1A* by Western blot (WB), then detected the mRNA expression of *PPARγ* via qRT-PCR to assess the regulatory relationship between *BMPR1A* and *PPARγ*.

WB results confirmed that 2.5 μM Dorsomorphin significantly reduced *BMPR1A* protein expression (p<0.01) compared with the control and DMSO groups, validating the effective inhibition of the target receptor by the inhibitor. Subsequently, qRT-PCR analysis showed that *PPARγ* mRNA abundance was markedly decreased in the Dorsomorphin-treated group relative to controls (p<0.05; Figures 7E, 7F). These *in vitro* data directly demonstrate that *BMPR1A* exerts a positive regulatory effect on *PPARγ* expression, filling the critical logical link between *BMPR1A* and adipogenesis (IMF deposition) and compensating for the limitations of computational virtual prediction. Collectively, the integration of virtual KO and *in vitro* validation confirms that *BMPR1A* acts as a key regulator of BMP signaling during bovine IMF deposition by modulating the expression of the core adipogenic factor *PPARγ*.

### Resveratrol-responsive cellular subpopulations exhibit distinct metabolic reprogramming signatures

**Figure 8:**
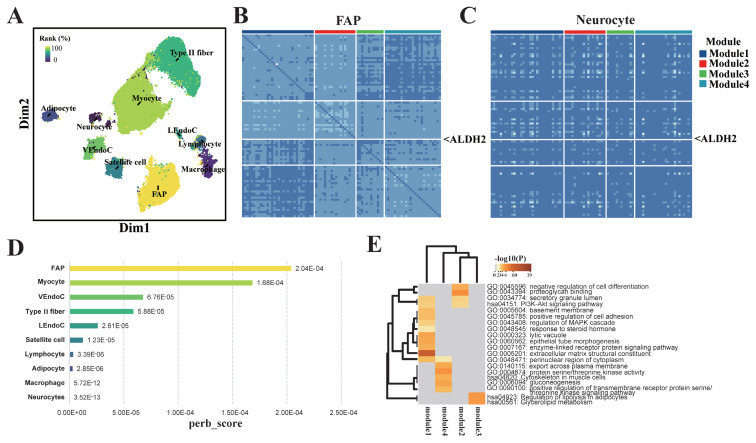
Cellular sensitivity to resveratrol. (A) UMAP plot of ranked sensitivity to resveratrol. (B) Modular heatmap of the top-ranked nerve cell. (C) Module heatmap of the last ranked FAPs. (D) Bar graph of perturbation scores for 10 cell types. (E) Heatmap of 4-module gene enrichment analysis. FAP, fibro/adipogenic progenitor cell.

Owing to the inherent heterogeneity among cell populations, responses to pharmacological agents such as resveratrol vary significantly across cell types. Thus, precise identification of drug-responsive cellular subsets is essential for elucidating mechanisms of drug action. Here, we characterized the hierarchical and molecular profiles of resveratrol-responsive cells within the skeletal muscle microenvironment using scRank, a computational method for single-cell drug response mapping. Our analysis revealed a distinct spatial segregation of cell clusters in UMAP visualization: FAPs (yellow cluster) and neurocytes (purple cluster) localized at opposing poles, with the remaining cell types distributed in intermediate regions (Figure 8A). Based on perturbation scores computed by scRank, neurocytes we identified as the most resveratrol-sensitive population (score = 2.04E-04), whereas FAPs were the least sensitive (score = 3.52E-13) (Figure 8B). Heatmap analysis further resolved cell type-specific regulatory dynamics. In both neurocytes and FAPs, genes were grouped into four co-expression modules: Module 1 contained 32 genes, Module 2 contained 17 genes, Module 3 contained 13 genes (including the target gene *ALDH2*), and Module 4 contained 25 genes (Figures 8C, 8D). Functional enrichment analysis suggested that Module 1 genes were primarily associated with extracellular matrix structural composition, epithelial tube morphogenesis, lytic vacuole function, and enzyme-linked receptor protein signaling pathways. Module 2 genes were enriched in proteoglycan binding, negative regulation of cell differentiation, secretory granule lumen, and the PI3K-Akt signaling pathway. Module 3 genes participated in the regulation of lipolysis in adipocytes and glycerolipid metabolism. Module 4 genes were involved in protein serine/threonine kinase activity, export across the plasma membrane, and the gluconeogenesis pathway (Figure 8E). Taken together, these computational analyses suggest that neurocytes may serve as the primary cellular targets of resveratrol, potentially coordinating lipid metabolic reprogramming through *ALDH2*-mediated mechanisms.

## DISCUSSION

IMF deposition is a critical determinant of beef quality, directly influencing juiciness, tenderness, and flavor. In this study, an integrated analysis of snRNA seq and bulk transcriptome data from ANG, HFD, and SIM cattle was performed to explore the cellular basis underlying breed-specific meat quality difference. Our data revealed substantial cellular heterogeneity among breeds. The predominance of myocytes (46.7%) and FAPs (26%) within ANG cattle skeletal muscle suggests a potential synergistic cellular environment conducive to IMF deposition. Does IMF originate from the transdifferentiation of the myogenic lineage or from the classical differentiation of intrinsic FAPs? Prior studies proved myoblasts can transdifferentiate into adipocytes. Yeow et al. [31] showed C2C12 cells convert to fat when myogenesis is blocked (DN MKK3) and *PPARγ* is activated (rosiglitazone) . Wang and Shan [32] reviewed multiple triggers: MyoD deletion, *PPARγ* overexpression, and aging. The existence of *PPARΓ ^+^/MYOD1^+^* double-positive cells confirmed the potential of myoblast transdifferentiation in bovine skeletal muscle, and the rare number and non-significant breed distribution of these cells further indicated that IMF deposition is not determined by transdifferentiation potential alone, but by the synergy of potential and VEndoCs-mediated microenvironmental regulation. Conversely, the skeletal muscle of HFD cattle was characterized by a glycolytic phenotype, dominated by type II fiber (54.3%). This cellular composition may be associated with a rapid post-slaughter pH decline, a phenomenon linked to reduced water-holding capacity and toughened meat [33]. Metabolic profiling using scFEA further indicated divergent metabolic states between breeds, with ANG samples showing higher inferred activity in modules related to glycolysis, the TCA cycle, and fatty acid metabolism. This could reflect an enhanced capacity to balance mitochondrial oxidative phosphorylation with anaerobic glycolysis, which might support efficient marbling.

Vasculogenesis provides oxygen and nutrients for adipocyte growth and deposition, and VEndoCs is the main component of the vasculature responsible for the delivery of oxygen, glucose, and other nutrients and metabolites, and can regulate the entry of glucose into skeletal muscle through vasodilatation or insulin transport [34]. Skeletal muscle homeostasis is known to be modulated by BMP signaling, and abnormal BMPR function may lead to skeletal and skeletal muscle diseases. As established in the literature and consistent with our mechanistic framework, *BMP2* and *BMP4* promote the predifferentiation of preadipocytes into mature adipocytes, thereby enhancing subcutaneous fat accumulation and marbling deposition across different developmental stages throughout the cattle rearing period. These ligands act as upstream signaling molecules that bind to and activate BMP type I receptors, initiating downstream adipogenic regulatory cascades. Studies in human fetal skeletal muscle have shown that BMP signaling is important for the proliferation of mesenchymal progenitor cells co-expressing *MYF5* and *BMPR1A* [35]. Endothelial cells are also capable of inhibiting muscle adipogenesis *in vivo* via BMP under physiological conditions [36]. It has been shown that mice with selective ablation of *BMPR1A* have more fat deposition, which is highly consistent with our observation of an endothelium-specific KO phenotype. In our study, hdWGCNA of VEndoCs identified yellow and brown gene modules enriched for BMP signaling components, which hints at a potential role for this pathway in modulating vascular function and adipogenic crosstalk within the muscle niche. *PPARγ* is a known major regulator of adipogenesis, and we conducted *in vitro* validation experiments using bovine skeletal muscle endothelial primary cells treated with 2.5 μM Dorsomorphin (BMPR1A inhibitor). The results showed that inhibiting *BMPR1A* activity significantly reduced the expression of *PPARγ* at the transcriptional and translational levels, indicating a positive regulatory relationship between *BMPR1A* and *PPARγ*. This finding is consistent with previous work [37], which directly links BMP signaling with upregulation of *PPARγ*, further confirming that *BMPR1A* plays a regulatory role in IMF deposition by regulating *PPARγ* expression. It is worth noting that virtual KO analysis did not identify *PPARγ* as the affected gene, which may be due to limitations in computer prediction. *In vitro* validation experiments effectively supplemented this deficiency and strengthened the reliability of our conclusion that *BMPR1A* is a key regulatory factor in IMF deposition. Furthermore, the inferred bidirectional activity of the PTPRM signaling pathway in VEndoCs points to a possible, tyrosine phosphatase-mediated layer of regulation that may fine-tune growth factor sensitivity and endothelial cell junction stability. We hypothesize that PTPRM-mediated signaling in VEndoCs may act as a homeostatic rheostat: by balancing tyrosine phosphorylation–dependent signaling events, it could stabilize endothelial cell junctions, modulate the responsiveness of VEndoCs to microenvironmental cues, and fine-tune the secretion or activity of paracrine factors (e.g., BMP ligands) that govern adipogenic commitment in the muscle niche. Notably, this vascular-metabolic interplay receives support from the scFEA predicted enrichment of glucose metabolism in VEndoCs, potentially correlating with enhanced nutrient flux that could support lipid droplet expansion in adjacent adipocytes. VEndoCs abundance varied among breeds, being highest in ANG cattle (4.1%) and lowest in SIM cattle (2.8%), which may suggest a positive association between endothelial cell prevalence and IMF deposition. Finally, our *in silico* perturbation analysis indicated *BMPR1A* as a key regulator within this network, as its computational knockdown was predicted to dysregulate13 downstream targets, including the acetyl coenzyme A carboxylase β (*ACACB*) and the RING-finger protein 3 gene containing the PDZ structural domain (*PDZRN3*).

Acetyl coenzyme A carboxylase (*ACC*) acts as a rate-limiting enzyme in fatty acid synthesis, catalyzing the conversion of acetyl coenzyme A to malonyl coenzyme A. This enzyme is involved in regulating fat deposition through a dual mechanism that includes the inhibition of β-oxidation [38,39]. The *CACAB* gene catalyzes the carboxylation of acetyl coenzyme A to malonyl coenzyme A, which is a major precursor for fatty acid synthesis. Notably, increased *ACACB* expression may also influence fatty acid oxidation by inhibiting carnitine palmitoyltransferase 1 (CPT-1) activity. For example, deep sequencing of Tibetan sheep skeletal muscle suggested that genes including *ACACB* could play important roles in lipid and fatty acid metabolism, potentially altering skeletal muscle energy metabolism through pathway such as AMPK signaling, which might affect meat quality [40]. Similarly, in the analysis of yak carcass and meat quality traits, genetic variation in *ACACB* has been proposed as a potential biomarker for meat quality improvement [41]. Studies in goats, beef cattle, pigs, and chickens have also indicated that the *ACACB* gene may be among the key factors influencing fat deposition [42–45]. Furthermore, it has been proposed that *PDZRN3* and its associated pathway could be important in the formation of intramuscular adipose tissue [43].

The identification of neuronal *ALDH2* as a resveratrol-responsive node in our analysis extends the investigation of the compound’s metabolic effects to a potential neurovascular regulatory axis within muscle. It should be noted that this resveratrol-related analysis is hypothesis-generating rather than confirmatory, as it is based on *in silico* predictions and preliminary associations rather than direct *in vitro* or *in vivo* experimental validation. Resveratrol is a naturally occurring polyphenolic compound that has received attention for its anti-obesity properties. Studies have shown that resveratrol can reduce obesity by improving insulin resistance and regulating lipid deposition via the PI3K/AKT signaling pathway, and by promoting white fat browning via the AMPK/SIRT1/PGC-1α signaling pathway [46]. In livestock studies, adding moderate amounts of resveratrol to diets improved meat quality, in contrast to the effects in humans. Zhang et al [47] added 600 mg/kg of resveratrol to the diet of pigs and fed it for 119 days, and found that pork quality, lipid profile, and IMF levels were significantly enhanced. The addition of resveratrol to the diet was found in goat meat quality studies increased redness and IMF content, whilst reducing shear force in the LD muscle of goats [48]. The *ALDH2* gene, a target of resveratrol, has been well-studied for its role in human liver disease, obesity, and aging, and in a genome-wide association study of obese individuals, *ALDH2* was found to play a key role in adipocyte function [49]. Yu’s study suggests that the *ALDH2* gene plays a key role in fat deposition. The study observed differences in *ALDH2* protein levels in adipose tissue of mice in different states, as well as the effects of *ALDH2* expression changes and activators on adipocyte differentiation [50]. Collectively, these prior findings provide a context for our computational result, which posits *ALDH2* as a potential neurovascular mediator of resveratrol’s action. This connection suggests a possible mechanistic bridge between the compound’s role in improving livestock meat quality and its broader metabolic effects, highlighting *ALDH2* as a candidate target worthy of further investigation across species.

Although this study provides a wealth of new insights into the cellular basis of IMF, there are still certain limitations: the small sample size (n = 2 per breed, with n = 1 for SIM cattle) limits the statistical power of the results and specifically impacts the robustness of breed-specific quantitative comparisons. Specifically, the observed breed differences in VEndoCs abundance, skeletal muscle metabolic activity, and paracrine signaling pathway dominance should be interpreted as biological trends rather than definitive quantitative rankings, as limited biological replication cannot rule out individual animal variation within each breed. While our study focused on the LD, the standard and economically critical site for marbling evaluation, we acknowledge that IMF deposition exhibits muscle-specific characteristics, and sampling from a single muscle limits the generalizability of our findings to other skeletal muscles (e.g., shank, hindquarter muscles). Future studies will expand sampling to multiple representative muscles with varying IMF content, explore muscle-specific regulatory mechanism of marbling. Additionally, the current snRNA-seq data cannot definitively distinguish the dominant pathway of IMF deposition between myoblast transdifferentiation and FAP *de novo* adipogenesis, which requires *in vivo* lineage tracing experiments for further validation. Although we have completed *in vitro* validation of *BMPR1A* function using bovine skeletal muscle endothelial primary cells and confirmed its positive regulatory effect on *PPARγ* at the transcription and translation levels through qRT-PCR and WB analysis, further validation is still needed to strengthen this conclusion, especially *in vivo* experimental validation, such as *BMPR1A* KO or overexpression animal models to verify its regulatory effect on IMF deposition at the tissue level, as well as cross breed validation to confirm species-specific regulatory patterns. We also note that snRNA-seq analysis of skeletal muscle tissue inherently carries potential technical artifacts and alternative interpretive considerations specific to this tissue type, including nuclear isolation challenges due to the large, multinucleated nature of muscle fibers, potential capture of fragmented nuclear material from disrupted cells, and mild cytoplasmic RNA contamination despite strict quality control.

In the future, we plan to expand sampling to multiple representative muscles with varying IMF content to explore muscle-specific regulatory mechanisms of marbling. We will also combine spatial transcriptome technology with an expanded sample size (≥3 biological replicates per breed) to further analyze and validate the spatial relationship between VEndoCs, adipocytes, and neural elements in the muscle microenvironment, as well as the breed-specific quantitative characteristics of key cell populations. Furthermore, future studies will expand the analytical scope to include multivariate regression analysis with a large cohort of samples, incorporating detailed nutritional (e.g., dietary fatty acid composition, energy intake) and environmental covariates to quantify their complex correlations with marbling accumulation capacity and fatty acid profiles (e.g., Omega-3, monounsaturated fatty acids). Additionally, our identification of the VEndoC-BMPR1A-IMF regulatory axis provides a molecular foundation for targeted nutritional interventions in cattle breeding. For example, dietary supplementation with Omega-3 or monounsaturated fatty acids, which are known *PPARγ* ligands, may synergize with the *BMPR1A*-mediated signaling pathway to enhance IMF deposition and improve marbling quality. Finally, we will integrate non-invasive ultrasound technology for *in vivo* marbling evaluation and EBV/EPD genetic evaluation tools to assist precise slaughter management and targeted muscle sampling, bridging genetic merit, phenotypic traits, and molecular mechanisms to facilitate the translation of our findings into practical applications for beef quality improvement and precision livestock breeding.

## CONCLUSION

In conclusion, our snRNA seq analysis identified VEndoCs as the core signaling hub regulating bovine IMF deposition, and scTenifoldKnk virtual KO prioritized *BMPR1A* as a key regulatory gene in the BMP pathway. *In vitro* validation further confirmed that *BMPR1A* positively regulates *PPARγ* expression, filling the logical gap between *BMPR1A* and adipogenesis. The integration of multiple computational analyses and *in vitro* validation provides new insights into the cellular and molecular mechanisms of IMF deposition in beef cattle, with potential translational value for improving beef quality.

## Figures and Tables

**Table 1:** Primer sequences for qRT-PCR

Primer name	Primer sequence
GADPH	AGCGAGATCCTGCCAACATCAAG
	GCAGGAGGCATTGCTGACAATCT
PPARγ	GAACCCAGAGTCTGCTGACC
	CGTGCACGCCGTATTTTAGG

qRT-PCR, quantitative real-time polymerase chain reaction.

## Data Availability

Upon reasonable request, the datasets of this study can be available from the corresponding author.

## SUPPLEMENTARY MATERIAL

**Supplement 1.** Comparison of evaluation metrics for different batching methods.

**Supplement 2.** Stacking diagram of cell proportions for five individuals.

**Supplement 3.** Identification of PPAR γ^+^/MyoD^+^ Double Positive Cells.

**Supplement 4.** The results of gene virtual knockout.

**Supplement 5.** Capacity for intercellular communication among skeletal muscles.

**Supplement 6.** River diagram of outgoing and incoming signal patterns related to cell populations of HFD and SIM breeds.

**Supplement 7.** Statistics on the positivity rate of marker genes.
