# Supplementary material for: Integrating single-nucleus RNA sequencing and gene perturbation reveals regulatory networks of intramuscular fat deposition in bovine skeletal muscle
Source: Anim Biosci. 2026 Mar 11;39(8):250897. doi: 10.5713/ab.250897 (PMC13441230; doi:10.5713/ab.250897)
Supplement: Supplementary file 1 [file ab-250897-Supplement-1.pdf]

Supplement 1. Comparison of evaluation metrics for different batching methods.

Method	Bio conservation					Batch correction					Aggregate score		
	Isolated labels	KMeans NMI	Kmeans ARI	Silhouette lable	CLISI	Silhouette batch	ILISI	KBET	Graph connectivity	PCR comparision	Batch correction	Bio conservation	Total
X_combat	0.58	0.30	0.14	0.44	0.93	0.81	0.23	0.03	0.48	0.99	0.51	0.48	0.49
X_scVI	0.57	0.24	0.08	0.49	0.86	0.90	0.40	0.10	0.52	0.73	0.53	0.45	0.48
X_harmony	0.57	0.29	0.14	0.44	0.91	0.83	0.29	0.10	0.36	0.87	0.49	0.47	0.48
X_pca	0.56	0.26	0.10	0.46	0.88	0.85	0.333	0.17	0.43	0	0.36	0.45	0.41
