# Supplementary material for: Integrating single-nucleus RNA sequencing and gene perturbation reveals regulatory networks of intramuscular fat deposition in bovine skeletal muscle
Source: Anim Biosci. 2026 Mar 11;39(8):250897. doi: 10.5713/ab.250897 (PMC13441230; doi:10.5713/ab.250897)
Supplement: Supplementary file 2 [file ab-250897-Supplement-2.pdf]

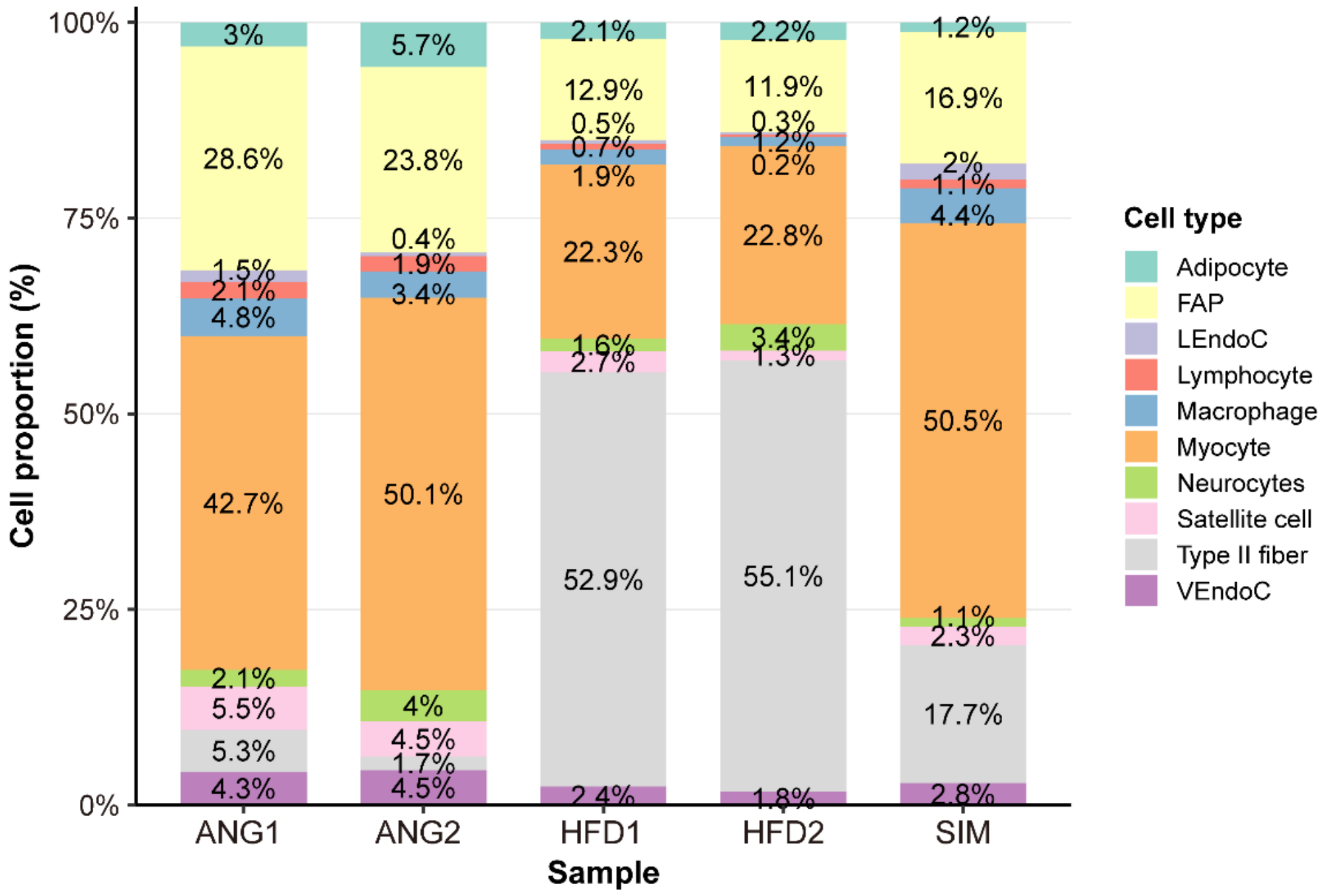

Supplement 2. Stacking diagram of cell proportions for five individuals.
