# Supplementary material for: Integrating single-nucleus RNA sequencing and gene perturbation reveals regulatory networks of intramuscular fat deposition in bovine skeletal muscle
Source: Anim Biosci. 2026 Mar 11;39(8):250897. doi: 10.5713/ab.250897 (PMC13441230; doi:10.5713/ab.250897)
Supplement: Supplementary file 3 [file ab-250897-Supplement-3.pdf]

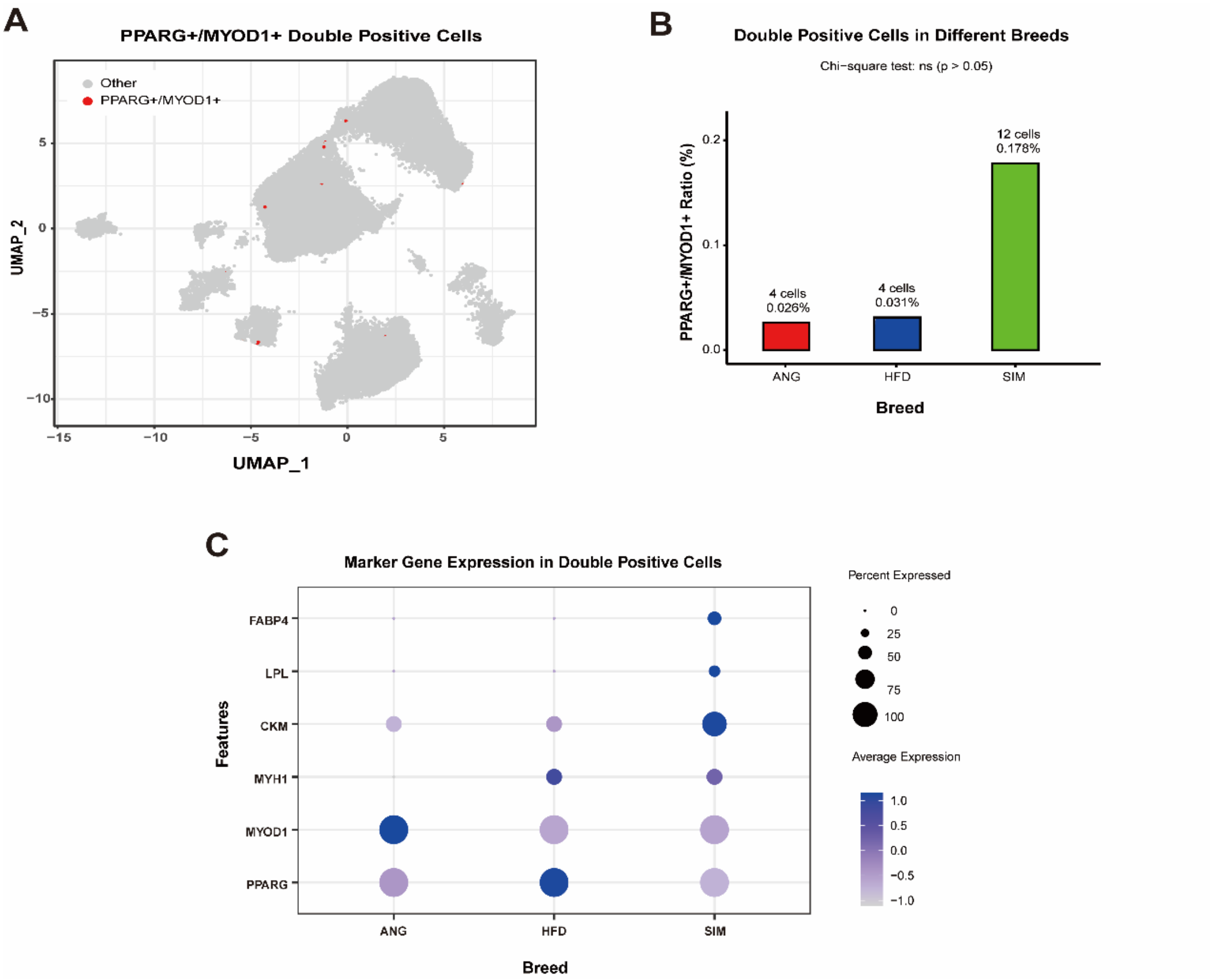

Supplement 3. Identification of PPAR γ $^{+}$/MyoD $^{+}$ Double Positive Cells. (A) Distribution of PPAR γ $^{+}$/MyoD $^{+}$ Double Positive Cells in UMAP. (B) Bar chart of the number and proportion of PPAR γ $^{+}$/MooD $^{+}$ double positive cells in different varieties. (C) Expression of myogenic and adipogenic marker genes in PPAR γ $^{+}$/MyoD $^{+}$ double positive cells.
