# Supplementary material for: Integrating single-nucleus RNA sequencing and gene perturbation reveals regulatory networks of intramuscular fat deposition in bovine skeletal muscle
Source: Anim Biosci. 2026 Mar 11;39(8):250897. doi: 10.5713/ab.250897 (PMC13441230; doi:10.5713/ab.250897)
Supplement: Supplementary file 4 [file ab-250897-Supplement-4.pdf]

Supplement 4. The results of gene virtual knockout.

Gene	Distance	boxcox-transformed distance	Z	FC	p-value	adjusted p-value	knockdown gene
MYH2	5.09E-06	-0.080931674	-6.175698765	802.3514967	0	0	ACVR1
TNNC2	5.06E-06	-0.080887057	-6.170005942	791.7794534	0	0	ACVR1
TNNC2	5.06E-06	-12.16978891	2.675473429	756.917525	0	0	ACVR2A
MYH2	5.09E-06	-12.1631868	2.67638693	767.0201327	0	0	ACVR2A
TNNC2	2.02E-05	-10.39725952	2.27303854	567.6310991	0	0	BMP6
MYH2	2.04E-05	-10.39111975	2.27390422	575.2176242	0	0	BMP6
ACACB	4.11E-08	-0.047354431	-4.964358187	69.13929029	1.11E-16	4.25E-13	BMPR1A
PDZRN3	3.36E-08	-0.046683358	-4.841202595	46.40893327	9.60E-12	2.45E-08	BMPR1A

EYA4	3.23E-08	-0.046548804	-4.816509146	42.79494595	6.08E-11	1.16E-07	BMPR1A
NEB	3.01E-08	-0.046320265	-4.774567556	37.25655711	1.04E-09	1.59E-06	BMPR1A
LDB3	2.99E-08	-0.046298195	-4.770517256	36.75904231	1.34E-09	1.71E-06	BMPR1A
TACC2	2.56E-08	-0.045792122	-4.677642399	26.92840006	2.11E-07	0.000231131	BMPR1A
ACTN2	2.49E-08	-0.045705154	-4.661682091	25.51150824	4.40E-07	0.000421228	BMPR1A
CTNNA3	2.43E-08	-0.045616659	-4.645441482	24.14202356	8.95E-07	0.000761924	BMPR1A
ENSBTAG00000046838	2.34E-08	-0.045501089	-4.624231845	22.45762646	2.15E-06	0.001646248	BMPR1A
MYOM1	2.33E-08	-0.045481366	-4.620612284	22.18147484	2.48E-06	0.001728046	BMPR1A
ENSBTAG00000053626	2.27E-08	-0.045401835	-4.606016768	21.10005275	4.36E-06	0.002783688	BMPR1A

RYR1	2.16E-08	-0.045250677	-4.578276242	19.18022678	1.19E-05	0.007011	BMPR1A
ADCY2	1.94E-08	-0.044908238	-4.51543164	15.42225753	8.60E-05	0.047056146	BMPR1A
TNNC2	1.01E-05	-0.085873274	-5.083492035	503.7709503	0	0	BMPR2
MYH2	1.02E-05	-0.08592343	-5.088451487	510.4971922	0	0	BMPR2
MYH2	1.02E-05	-11.26014551	2.546975408	712.103633	0	0	SMAD1
TNNC2	1.01E-05	-11.26650885	2.546043572	702.7196675	0	0	SMAD1
MYH2	5.09E-06	-0.079920043	-6.122869249	963.7938927	0	0	SMAD2
TNNC2	5.06E-06	-0.079875421	-6.117300328	951.0910005	0	0	SMAD2
TNNC2	1.01E-05	-0.085869605	-5.019410902	447.8164489	0	0	SMAD6
MYH2	1.02E-05	-0.085919725	-5.024344715	453.7912054	0	0	SMAD6
