# Supplementary material for: Integrating single-nucleus RNA sequencing and gene perturbation reveals regulatory networks of intramuscular fat deposition in bovine skeletal muscle
Source: Anim Biosci. 2026 Mar 11;39(8):250897. doi: 10.5713/ab.250897 (PMC13441230; doi:10.5713/ab.250897)
Supplement: Supplementary file 5 [file ab-250897-Supplement-5.pdf]

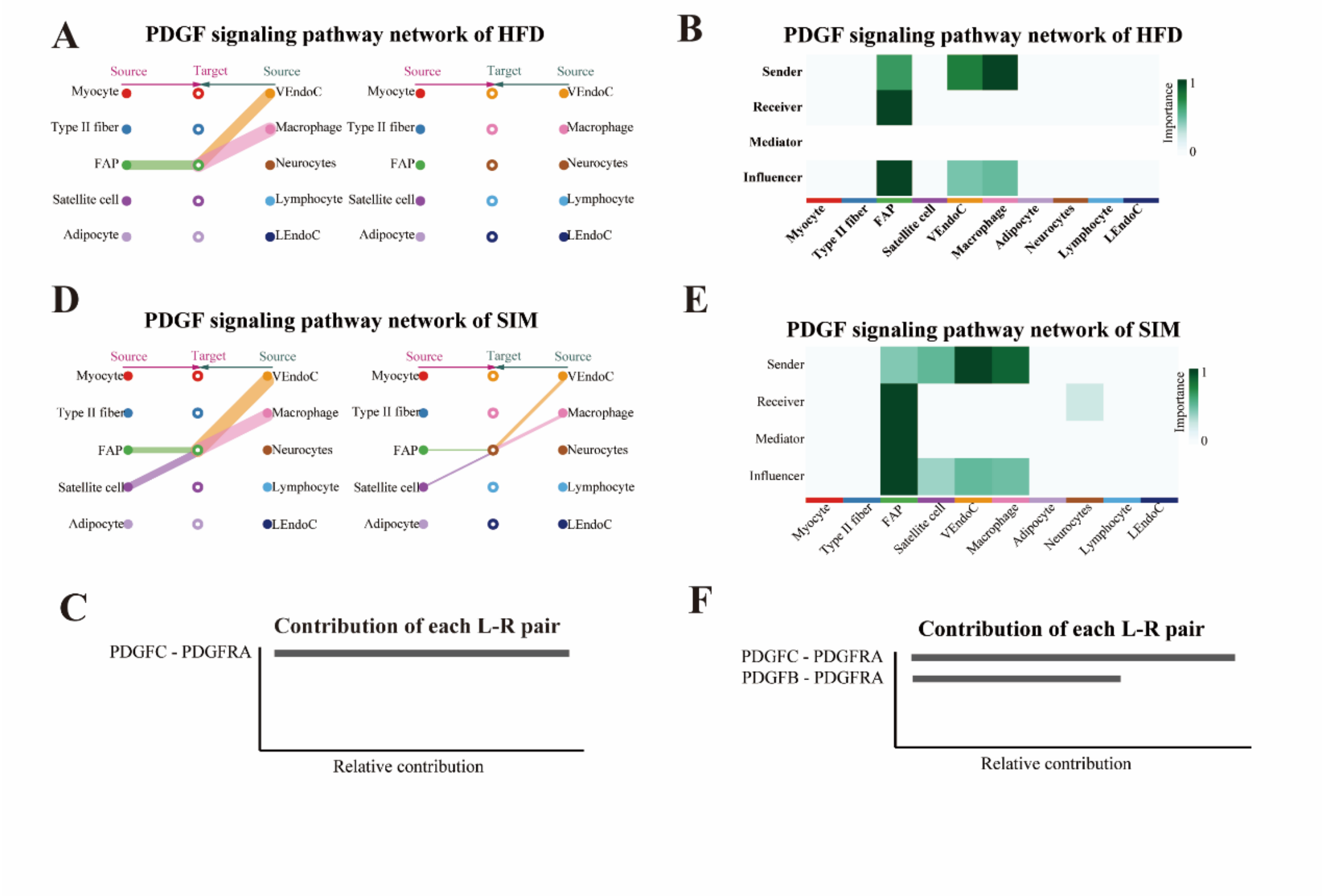

Supplement 5. Capacity for intercellular communication among skeletal muscles. (A) Hierarchical diagram of the PDGF pathway of HFD cattle. Lines represent intercellular interactions, and their thickness is proportional to the communication probability of the cell interaction. Line colors are consistent with the signaling source. (B) Heatmap shows the role (sender, receiver, mediator or influencer) of each cell group in PDGF signaling of HFD cattle. Color saturation indicates the importance of signaling role in the pathway network. (C) Relative contribution of ligand-receptors of the PDGF pathway to the overall signaling network. (D) Hierarchical diagram of the PDGF pathway of SIM cattle. (E) Heatmap shows the role (sender, receiver, mediator or influencer) of each cell group in PDGF signaling of SIM cattle. (F) Relative contribution of ligand-receptors of the PDGF pathway to the overall signaling network.
