# Supplementary material for: Integrating single-nucleus RNA sequencing and gene perturbation reveals regulatory networks of intramuscular fat deposition in bovine skeletal muscle
Source: Anim Biosci. 2026 Mar 11;39(8):250897. doi: 10.5713/ab.250897 (PMC13441230; doi:10.5713/ab.250897)
Supplement: Supplementary file 6 [file ab-250897-Supplement-6.pdf]

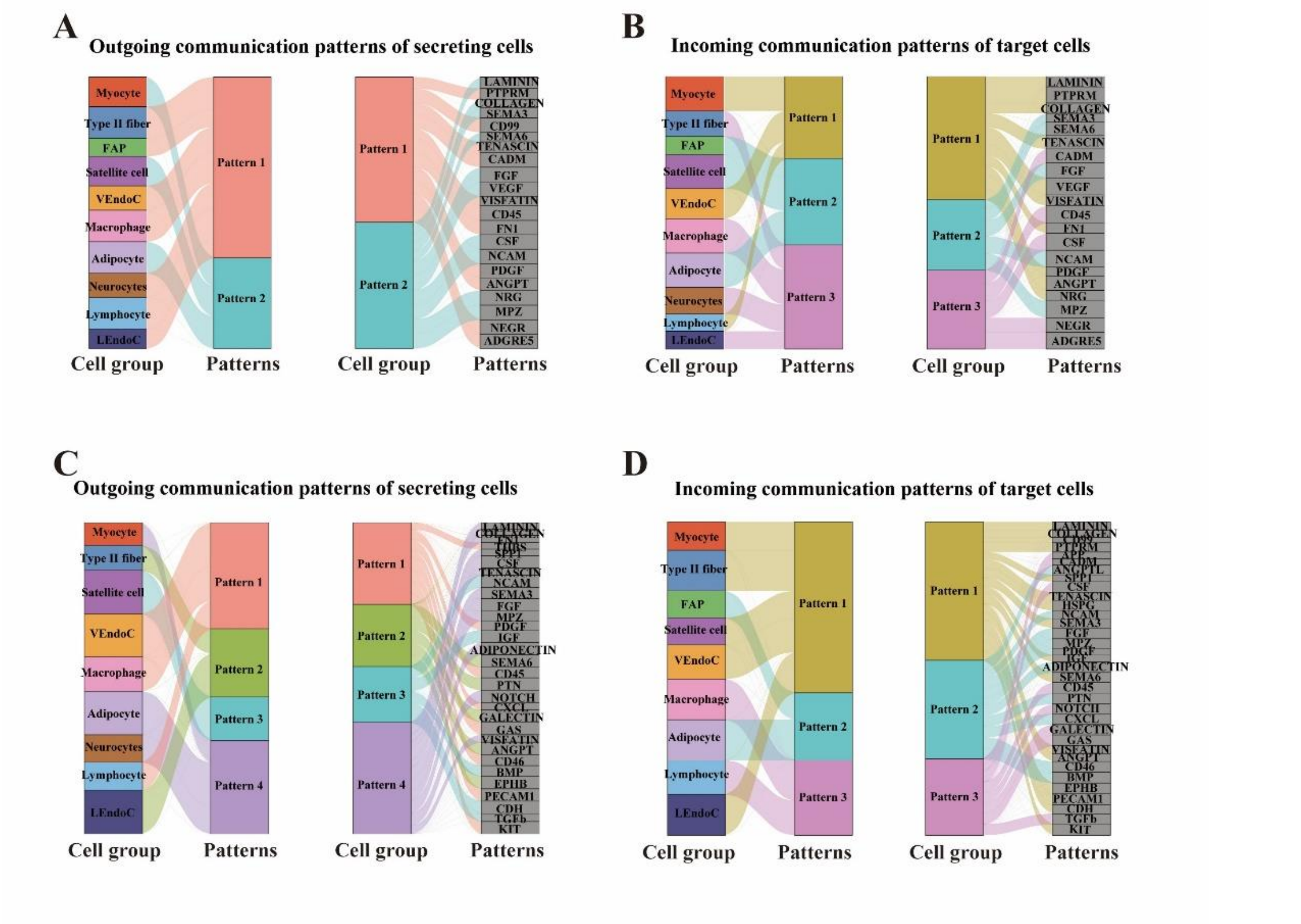

Supplement 6. (A) The outgoing communication patterns of secreting cells of HFD cattle. The flow thickness represents the contribution of a signaling pathway to each mode of communication. (B) The inferred incoming communication patterns of target cells of HFD cattle. (C) The outgoing communication patterns of secreting cells of SIM cattle. (D) The inferred incoming communication patterns of target cells of SIM cattle.
