# Supplementary material for: Integrating single-nucleus RNA sequencing and gene perturbation reveals regulatory networks of intramuscular fat deposition in bovine skeletal muscle
Source: Anim Biosci. 2026 Mar 11;39(8):250897. doi: 10.5713/ab.250897 (PMC13441230; doi:10.5713/ab.250897)
Supplement: Supplementary file 7 [file ab-250897-Supplement-7.pdf]

Supplement 7. Statistics on the positivity rate of marker genes.

**Marker**	PPARγ	MYOD1	MYH1	CKM	ACACB	FABP4
**Positivity rate(%)**	100	100	40	70	65	25
